# Characteristics of plant trait network and its influencing factors in impounded lakes and channel rivers of South-to-North Water Transfer Project, China

**DOI:** 10.3389/fpls.2023.1127209

**Published:** 2023-03-10

**Authors:** Tianshun Zhu, Wanxiang Jiang, Henglun Shen, Juanjuan Yuan, Jing Chen, Zheng Gong, Lihong Wang, Meng Zhang, Qingyang Rao

**Affiliations:** ^1^ College of Life Sciences, Zaozhuang University, Zaozhuang, China; ^2^ Institute of Aquatic Environment, Jiangxi Academy of Eco-Environmental Sciences and Planning, Nanchang, China; ^3^ Institute for Ecological Research and Pollution Control of Plateau Lakes, School of Ecology and Environmental Science, Yunnan University, Kunming, China

**Keywords:** traits variation, hub traits, network topology, total phosphorus, dissolved oxygen

## Abstract

Trait-based approaches have been widely used to evaluate the effects of variable environments on submerged macrophytes communities. However, little research focused on the response of submerged macrophytes to variable environmental factors in impounded lakes and channel rivers of water transfer project, especially from a whole plant trait network (PTN) perspective. Here, we conducted a field survey designed to clarify the characteristic of PTN topology among impounded lakes and channel rivers of the East Route of South-to-North Water Transfer Project (ERSNWTP) and to unravel the effects of determining factors on the PTN topology structure. Overall, our results showed that leaf-related traits and organ mass allocation traits were the hub traits of PTNs in impounded lakes and channel rivers of the ERSNWTP, which traits with high variability were more likely to be the hub traits. Moreover, PTNs showed different structures among impounded lakes and channel rivers, and PTNs topologies were related to the mean functional variation coefficients of lakes and channel rivers. Specially, higher mean functional variation coefficients represented tight PTN, and lower mean functional variation coefficients indicated loose PTN. The PTN structure was significantly affected by water total phosphorus and dissolved oxygen. Edge density increased, while average path length decreased with increasing total phosphorus. Edge density and average clustering coefficient showed significant decreases with increasing dissolved oxygen, while average path length and modularity exhibited significant increases with increasing dissolved oxygen. This study explores the changing patterns and determinants of trait networks along environmental gradients to improve our understanding of ecological rules regulating trait correlations.

## 1 Introduction

Plant functional traits are measurable properties which determine plant survival, growth and reproduction (Díaz et al., 1999; Westoby and Wright, 2006; Violle et al., 2007; Wright et al., 2010). Plant traits reflect a comprehensive process of evolutionary signal, species specificity, physiological function and environmental constraints (Palma et al., 2021; Guo et al., 2022; Laine et al., 2022). In natural lakes, submerged macrophytes traits variations caused by environment are nearly three times higher than that caused by ontogeny, and environmental filtering processes can sort individuals within species with traits values adaptive to environmental changes (Kraft et al., 2008; Fu et al., 2013).

Submerged macrophytes can change the morphology and biomass allocation of leaves, stems, and roots to support the survival of the species in response to different environmental changes (Fu et al., 2020; Wang et al., 2021). In the past decades, many studies mainly focused on the response of single or several submerged macrophytes traits to environmental gradients. For instance, *Potamogeton crispus* showed an increase in leaf length, leaf area and plant height and a decrease in leaf thickness and stem diameter along water depth from 0.4 m to 1.6m (Wang et al., 2021). *Vallisneria natans* displayed a decrease in ramets number, ramet biomass, root/shoot ratio and ramet/total biomass ratio with underwater light weakened (Yang et al., 2022).

For plants, many functional traits are dependent and intercorrelated, traits coordination is generally defined by negative and/or positive correlations, representing co-optimization, trade-offs, and allometries based on physiological, morphological, and evolutional requirements in response to surrounding environments (Wright et al., 2004; Kemppinen et al., 2021; Pothasin et al., 2022). Previous studies have shown that plant functional traits and their interactions and coordination are keys to understanding ecosystem processes and functions (Kleyer et al., 2019; Palma et al., 2021; Laine et al., 2022).

Recently, plant trait network (PTN) has been proved to be an effective approach to reveal complex correlations among plant traits, detect hub traits from suites of functional traits, and calculate the parameters of overall topology of traits combination (Proulx et al., 2005; Poorter et al., 2014; Kleyer et al., 2019; He et al., 2020; Li et al., 2021). For instance, contemporary studies on the impacts of climate on leaf trait networks (LTNs) pointed that LTNs changed from a complex and tight topology in tropical forest to a simple and loose structure in cold-temperate forest, and leaf thickness and leaf economic traits were hub traits in LTNs (Li et al., 2022). Currently, Rao et al. (2022) proposed that water total phosphorus concentration (TP) could alter the overall PTNs topology of submerged macrophyte and PTNs were loose in TP-deficiency and TP-repletion water but tight in TP-moderation water. Wang et al. (2022) pointed that submerged macrophyte PTNs structure was more dispersed under low or high nutrient levels than that found at moderate nutrient levels. Yuan et al. (2023) showed that ammonium pulses enhanced trait connectivity in submerged macrophyte PTNs, and the highly connected traits were plant biomass, stem ratio, leaf ratio and ramet number in PTNs, which were related to biomass allocation. Thus, PTNs can provide an integrative information about submerged macrophytes response to environmental changes in impounded lakes and channel rivers of water transfer project.

Here, we presented a field survey designed to examine variation of hub traits and PTNs topologies among channel rivers and impounded lakes of ERSNWTP and test the effects of determining factors on the PTNs topologies. We tested three questions: (1) How do functional traits vary among impounded lakes and channel rivers? (2) How do the hub traits and submerged macrophytes PTNs topologies vary among channel rivers and impounded lakes? (3) What are the key environmental factors that determine PTNs topologies?

## 2 Materials and methods

### 2.1 Study area

China’s South to North Water Transfer Project (SNWTP) is a world-famous hydraulic project, which aims to transfer water resource from southern part of China to the north and northwest regions. It involves water redistribution among different basins and may cause a series of changes in ecological conditions of impounded lakes and channel rivers ecosystems (Zeng et al., 2015; Guo et al., 2020; Sun et al., 2021). SNWTP consists of ERSNWTP, Central Route of South-to-North Water Transfer Project (CRSNWTP), and West Route of South-to-North Water Transfer Project (WRSNWTP). ERSNWTP pumps water from the Yangtze River in Yangzhou, Jiangsu Province, employs Lake Gaoyou, Hongze, Luoma, Nansi and Dongping for water impoundment, utilizes the Grand Canal and its parallel rivers to transfer water from south to north (http://nsbd.mwr.gov.cn). Nansi Lake was divided into upper and lower region by an artificial dam since 1960s, the water is first transported into the lower region and then is lifted and moved to upper region during water diversion. The morphometrical and limnological characteristics of these impounded lakes were detailed in **Table S1**.

### 2.2 Field survey and sampling

**Figure 1:**
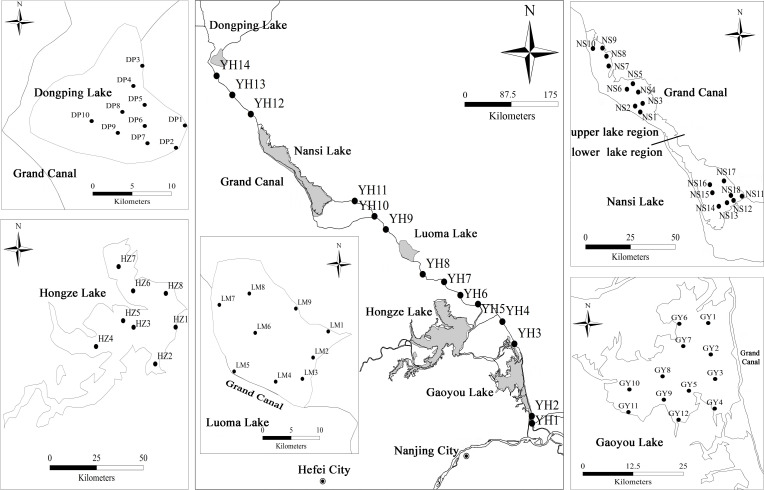
Sampling sites of impounded lakes and Grand Canal of the East Route of South-to-North Water Transfer Project.

*P. crispus* is dominant and constructive species of submerged macrophyte community in the impounded lakes and channel rivers of ERSNWTP in China during winter and spring (Zhu et al., 2022). *P. crispus* population were sampled in five impounded lakes and Grand Canal from 10 April to 12 May 2018 when the biomass of *P. crispus* reached its peak in these lakes. 74 sites were surveyed in *P. crispus*-dominated region using site-quadrat method. The number of sampling sites in impounded lakes and Grand Canal were determined based on the distribution area of *P. crispus*, 3 sampling sites were selected in every Grand Canal section between each two impounded lakes (**Figure 1**). *P. crispus* population were sampled using a rotatable reaping hook covering 0.2 m^2^, three replicated quadrats at each site. At each quadrat, the collected macrophytes were rinsed in lake water and then one healthy *P. crispus* individual was randomly selected for subsequent functional traits measurement. Prior to plant sampling, water and sediment samples were sampled from 0.5 m below the water surface using a Schindler sampler and the top layers of sediment using a Peterson sampler, respectively. These samples were kept in a container with ice bags and taken back to the laboratory for subsequent analysis.

For all sites, water pH, dissolved oxygen (DO), conductivity (Cond), total dissolved solid (TDS), turbidity (Tur), Secchi depth (SD) and underwater photosynthetic active radiation (PAR) were recorded *in situ*. T, DO, pH, Cond, TDS and Tur were measured with a YSI EXO2 (Yellow Springs Instruments, USA). SD was measured by Secchi disk (30 cm diameter). Underwater PAR was recorded at intervals of 0.25 m from water surface to 1.0 m by an underwater radiation sensor (UWQ 10250) coupled with a LI-COR data logger (Li-1500, Li-Cor Company, USA). The light extinction coefficient (K) was computed based on the equation: *K* =(1/*d*) ln (*I_0_*/*I_d_*), where *I_d_* and *I_0_* are PAR value at the water depth *d* and water surface (Krause-Jensen and Sand-Jensen, 1998).

### 2.3 Laboratory analysis and traits measurement

Water samples were used to measure total nitrogen (TN), total phosphorus (TP), ammonium nitrogen (NH_4_^+^-N), nitrate nitrogen (NO_3_^–^N), orthophosphate (PO_4_^3+^-P), permanganate index (COD_Mn_), and chlorophyll *a* (Chl *a*) in the laboratory according to the standard methods (Huang et al., 1999).

Sediment samples were used to analyze water content (S_W_), organic matter content (S_O_), total carbon content (S_C_), total nitrogen content (S_N_) and total phosphorous (S_P_). S_W_ and S_O_ were determined using the methods detailed by Jin and Tu (1990). Sediment samples were air dried to constant weight and then ground into fine powder for elemental determination. S_C_ and S_N_ were measured by an element analyzer (Multi N/C 2100, Jena Instruments, German). For S_P_ measurement, sediment samples were digested with sulfuric acid/perchloric acid and then measured with the ammonium molybdate ascorbic acid method (Huang et al., 1999).

Eighteen functional traits of 222 P*. crispus* individuals sampled in five impounded lakes and Grand Canal were measured during the study period: plant height (H), stem branch (SB), internodes number (NN), internode length (NL), internode diameter (ND), relative stem length (RSL), relative leaf area (RLA), leaf width (LW), leaf area (LA), leaf number (LN), leaf thickness (LT), leaf length (LL), dry plant weight (DPW), dry leaf weight (DLW), dry stem weight (DSW), ratio of stem weight to leaf weight (SLR), leaf dry mass fraction (LMF) and stem dry mass fraction (SMF). Details of the functional traits and determination methods were described in **Table 1**.

**Table 1:** Plant traits and their units, abbreviations, and determination methods.

Functional traits	Unit	Abbreviation	Determination method
Plant height	cm	H	Ruler measurement
Stem branch	–	SB	Continuous counting
Internodes number	–	NN	Continuous counting
Internode length	cm	NL	Ruler measure
Internode diameter	mm	ND	Vernier caliper measurement
Relative stem length	cm g^-1^	RSL	Plant height/Dry stem weight
Leaf number	–	LN	Continuous counting
Leaf thickness	mm	LT	Vernier caliper measurement
Leaf length	cm	LL	Leaf area analyzer (CI-202)
Leaf width	cm	LW	Leaf area analyzer (CI-202)
Leaf area	cm^2^	LA	Leaf area analyzer (CI-202)
Relative leaf area	cm^2^ g^-1^	RLA	Leaf area/Dry leaf weight
Dry plant weight	g	DPW	Electronic balances measurement
Dry stem weight	g	DSW	Electronic balances measurement
Dry leaf weight	g	DLW	Electronic balances measurement
Ratio of stem weight to leaf weight	–	SLR	Dry stem weight/Dry leaf weight
Stem dry mass fraction	–	SMF	Dry stem weight/Fresh stem weight
Leaf dry mass fraction	–	LMF	Dry leaf weight/Fresh leaf weight

### 2.4 Plant trait network analysis

Submerged macrophytes PTNs were constructed to reveal the difference in trait associations among impounded lakes and Grand Canal. In PTN, plant traits are nodes, and trait-trait connections are edges. First, a matrix of trait-trait coefficients was computed using Pearson’s correlation coefficients (Poorter et al., 2014; Kleyer et al., 2019). To avoid spurious relationships among traits, trait-trait coefficients were assigned as 1 if significant at *P*<0.05 level and regarded as 0 if insignificant. Then, an adjacency matrix A = [*a_i_*,*_j_*] with *a_i_*,*_j_* ∈ [0,1] was yielded. Thus, PTN only showed the presence and absence of correlations among traits. Next, PTNs were constructed and network parameters were calculated using the R package “igraph”. Finally, PTNs were visualized in Cytoscape 3.8.0 using the Prefuse Force Directed OpenCL layout (Shannon et al., 2003).

**Figure 2:**
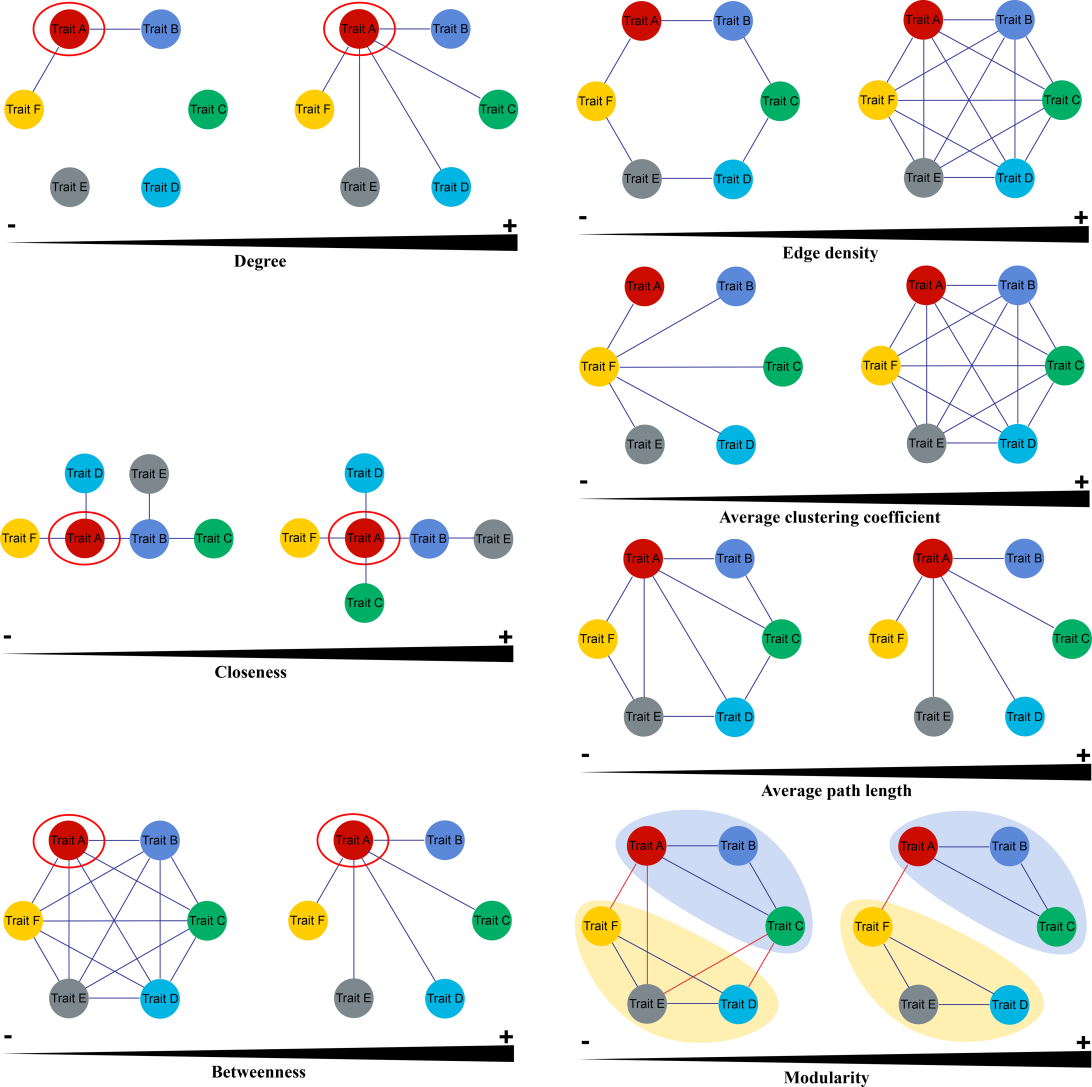
Key parameters of Plant Trait Network (Cited from He et al., 2020).

Three node parameters were selected to describe the properties of functional traits within PTNs. Degree (*k*) is calculated as the sum of the edges over all neighbors of the focal trait in the network. Plant traits with high *k* values can be regard as “hub traits”. Closeness (*C*) is the reciprocal of the mean shortest path between a focal trait and all other traits. Traits with high *C* values are closely associated with other traits. The betweenness (*B*) of a focal trait is determined as the number of shortest paths between pairs of traits that contain the focal trait. Traits that have a high *k* are generally considered as mediators in the PTNs (**Figure 2**).

Four metrics were used to show the overall topology of PTNs. Edge density (*ED*) represents the density of the connected edges among nodes in a network, that is, the proportion of actual correlations among traits out of all possible correlations. Average path length (*AL*) is the mean shortest path between all traits in the network. PTNs that have higher *AL* indicate greater overall independence among traits. Average clustering coefficient (*AC*) is the mean of the clustering coefficients of all node traits in PTNs. PTNs with higher *AC* are less easily separated into several different modules. Modules are defined as sets of traits that present covariation among themselves. Modularity (*Q*) measures how well a network is divided into modules. The trait network with higher modularity may give macrophytes more flexibility to adjust its functioning to changing environments (**Figure 2**). More details on network parameters were presented by He et al. (2020) and Kleyer et al. (2019).

### 2.5 Data analysis

To describe the importance of traits within PTNs, *k* and *C* were used to determine “the connectedness” of each trait, *B* was used to signify the “centrality” of each trait. Traits that have high *k* and high *C* were “hub traits”, and high *B* were “mediator traits”.

To describe the overall topology of PTNs, *ED* and *AL* were used to represent the “connectivity” of the PTN, *AC* and *Q* were used to characterize the “complexity” of the PTN. PTNs with low *ED* and high *AL* are “looser”, that is, they have an overall low level of covariation among traits. A high *AC* indicates fewer modules, and a lower PTN complexity. The *Q* of the PTN describes the degree of separation among modules.

One-way ANOVA with Duncan’s test (at the 0.05 significance level) was used to evaluate the difference of functional traits and also the environmental parameters among impounded lakes and Grand Canal. All data were tested for normality and homogeneity, when necessary, data were log_10_ and sqrt transformed to meet assumptions before statistical analysis. Linear regression was used to examine the relationships between traits variations and three node parameters and four topological metrics. To explore the determining environmental factors of PTNs, Pearson’s correlation was performed to detect the significant environmental factors related to parameters of PTNs, then one or multiple variable linear regression was computed using PTN topology metrics (i.e. *ED*, *AL*, *AC*, *Q*) as the dependent variable, and the significant environmental factors detected by Pearson’s correlation as the independent variables.

## 3 Results

### 3.1 Characteristic of environmental factors among different lakes

Cond, pH, DO, TDS, Tur, SD, K, Chl *a*, TN, NO_3_-N, NH_4_-N, TP, PO_4_-P, S_w_, S_o_, S_C_ and S_N_ differed significantly among impounded lakes and Grand Canal except COD_Mn_ and S_P_ (**Table S2**).

### 3.2 Variation of functional traits among different lakes

All 18 functional traits were significantly different among impounded lakes and Grand Canal (**Table S3**). In terms of the mean coefficient of variation (CV) of traits from high to low, the order of 18 functional traits was stem branch, dry leaf weight, relative leaf area, dry plant weight, leaf number, dry stem weight, internodes number, leaf thickness, ratio of stem weight to leaf weight, relative stem length, internodes length, plant height, leaf area, leaf dry mass fraction, stem dry mass fraction, leaf length, internode diameter, leaf width (**Table S4**). The results showed that leaf-related traits and organ mass allocation traits were sensitive traits that varying with various environment.

### 3.3 Identification of connected and central traits within PTNs

**Figure 3:**
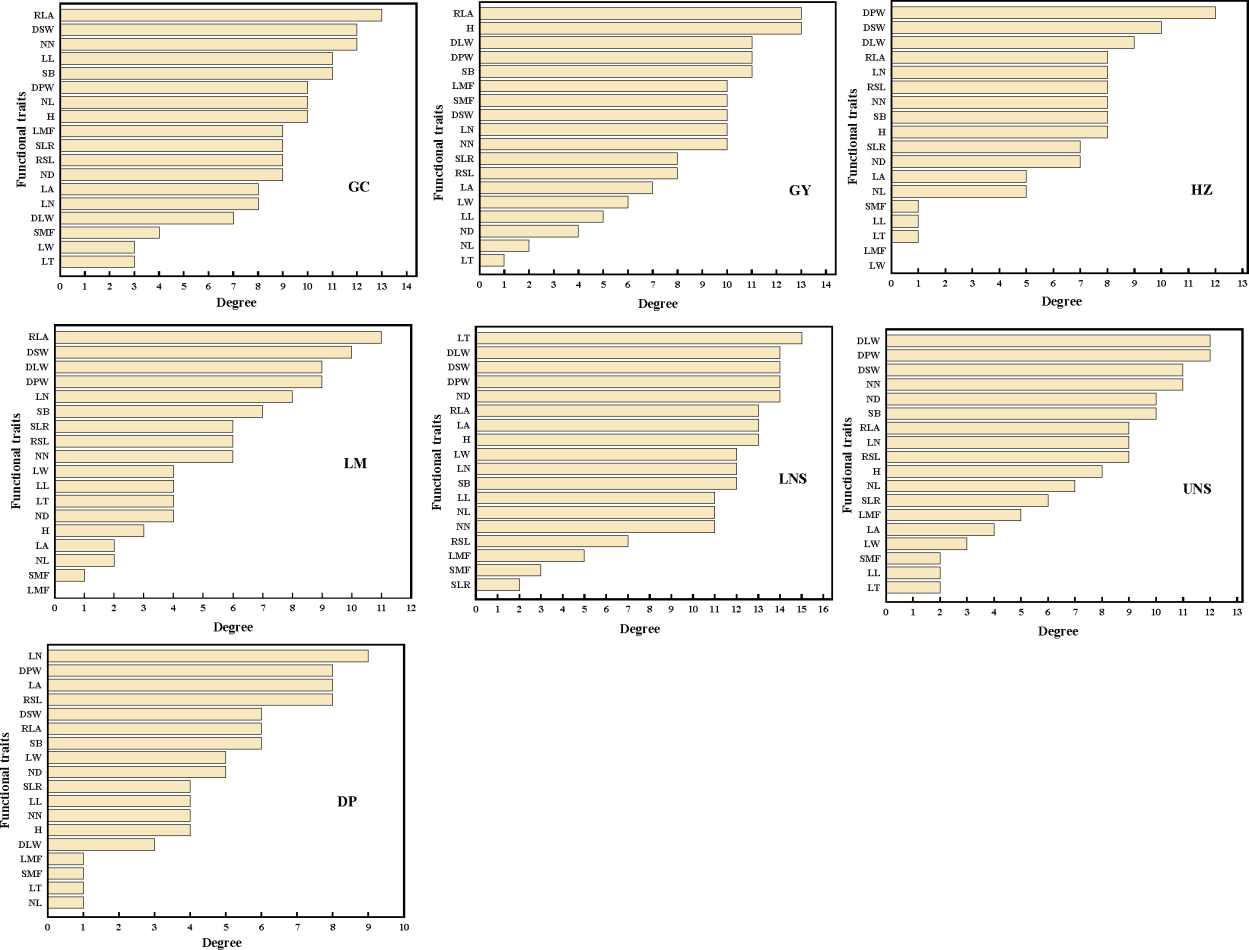
The degree of functional traits in plant trait network of impounded lakes and Grand Canal. GC, GY, HZ, LM, LNS, UNS and DP are abbreviations for Grand Canal, Gaoyou Lake, Hongze Lake, Luoma Lake, lower Nansi Lake, upper Nansi Lake and Dongping Lake, respectively.

**Figure 4:**
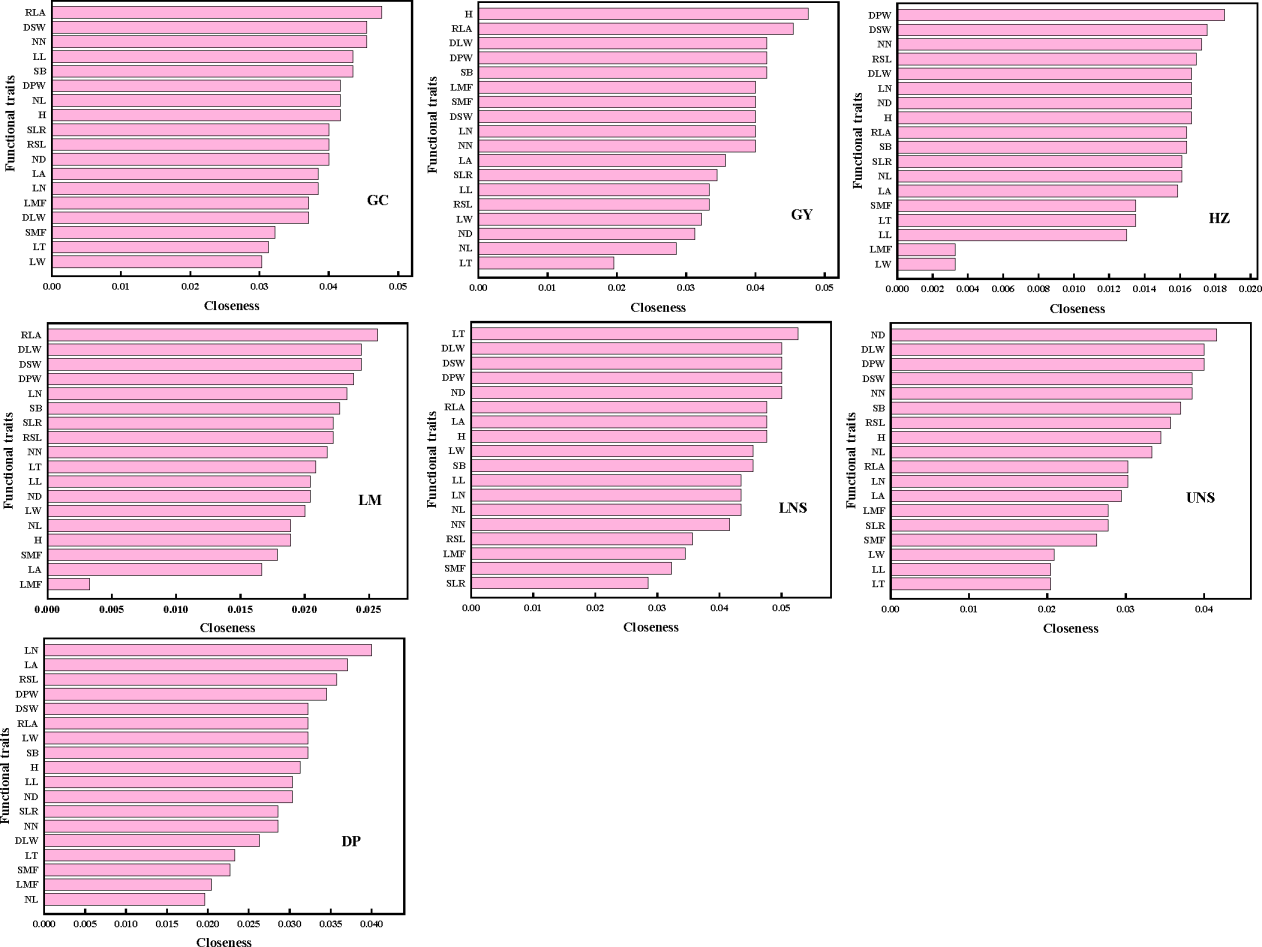
The closeness of functional traits in plant trait network of impounded lakes and Grand Canal. GC, GY, HZ, LM, LNS, UNS and DP are abbreviations for Grand Canal, Gaoyou Lake, Hongze Lake, Luoma Lake, lower Nansi Lake, upper Nansi Lake and Dongping Lake, respectively.

The most connected traits (with higher *k* and/or *C*) were relative leaf area, dry stem weight and internodes number in Grand Canal; relative leaf area, plant height, dry plant weight, stem branch and dry leaf weight in Gaoyou Lake; dry plant weight, dry stem weight and internodes number in Hongze Lake; relative leaf area, dry stem weight and dry leaf weight in Luoma Lake; leaf thickness, dry plant weight, dry leaf weight, dry stem weight and internode diameter in lower Nansi Lake; dry plant weight, dry leaf weight, dry stem weight, internodes number and internode diameter in upper Nansi Lake; and leaf number, dry plant weight, leaf area and relative stem length in Dongping Lake (**Figures 3**, **4**). These results indicated that seven natural *P. crispus* L. populations showed different properties of functional traits within PTNs among impounded lakes and Grand Canal.

**Figure 5:**
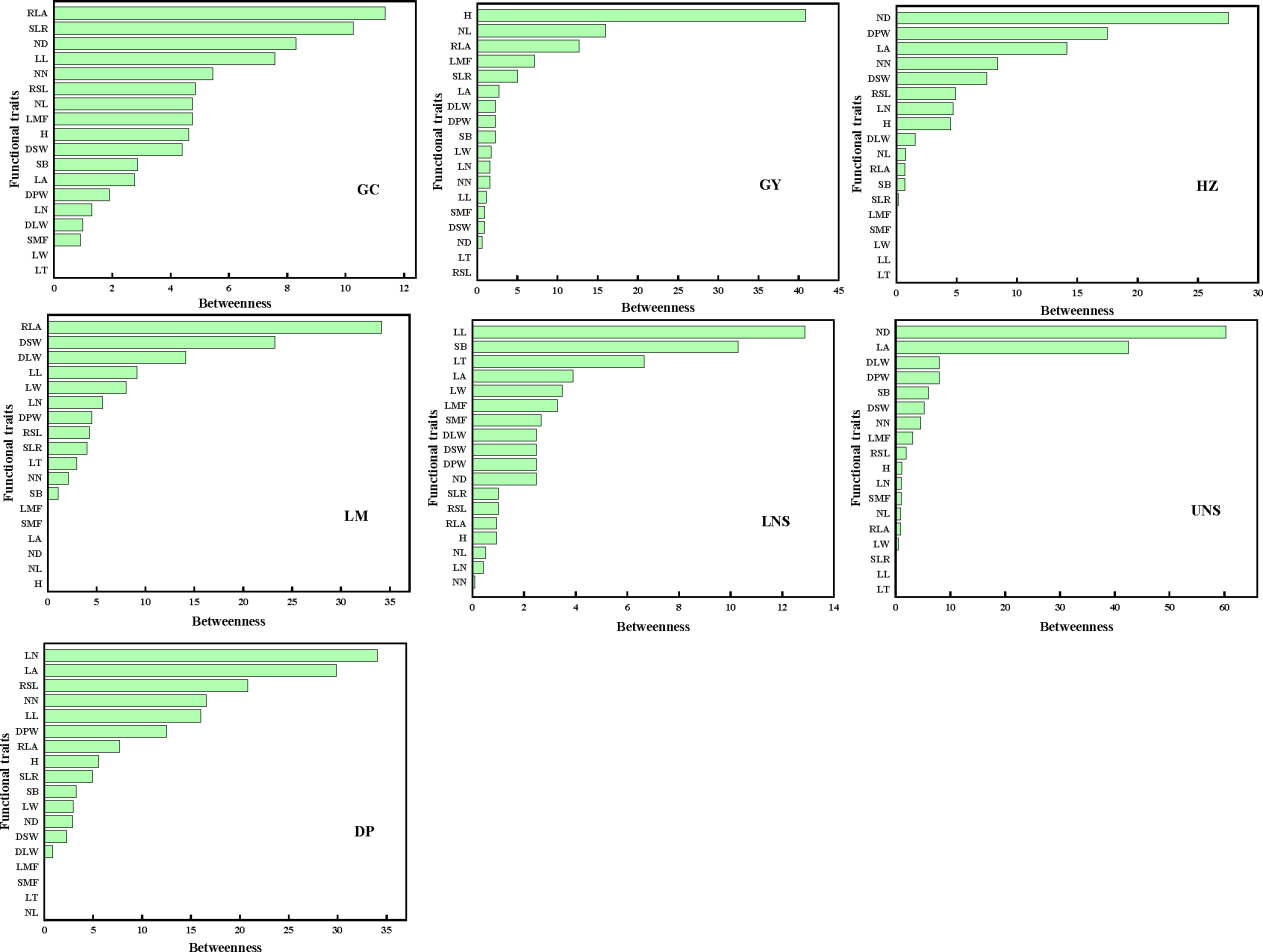
The betweenness of functional traits in plant trait network of impounded lakes and Grand Canal. GC, GY, HZ, LM, LNS, UNS and DP are abbreviations for Grand Canal, Gaoyou Lake, Hongze Lake, Luoma Lake, lower Nansi Lake, upper Nansi Lake and Dongping Lake, respectively.

The mediator traits (with high *B*) were relative leaf area and ratio of stem weight to leaf weight in Grand Canal; plant height, internode length and relative leaf area in Gaoyou Lake; internode diameter, dry plant weight and leaf area in Hongze Lake; relative leaf area, dry stem weight and dry leaf weight in Luoma Lake; leaf length and stem branch in lower Nansi Lake; internode diameter and leaf area in upper Nansi Lake; and leaf number and leaf area in Dongping Lake (**Figure 5**). Such traits acted as bridges in the PTNs by connecting other traits belonging to different modules.

### 3.4 The characteristics of PTNs topologies among impounded lakes/Grand Canal

**Figure 6:**
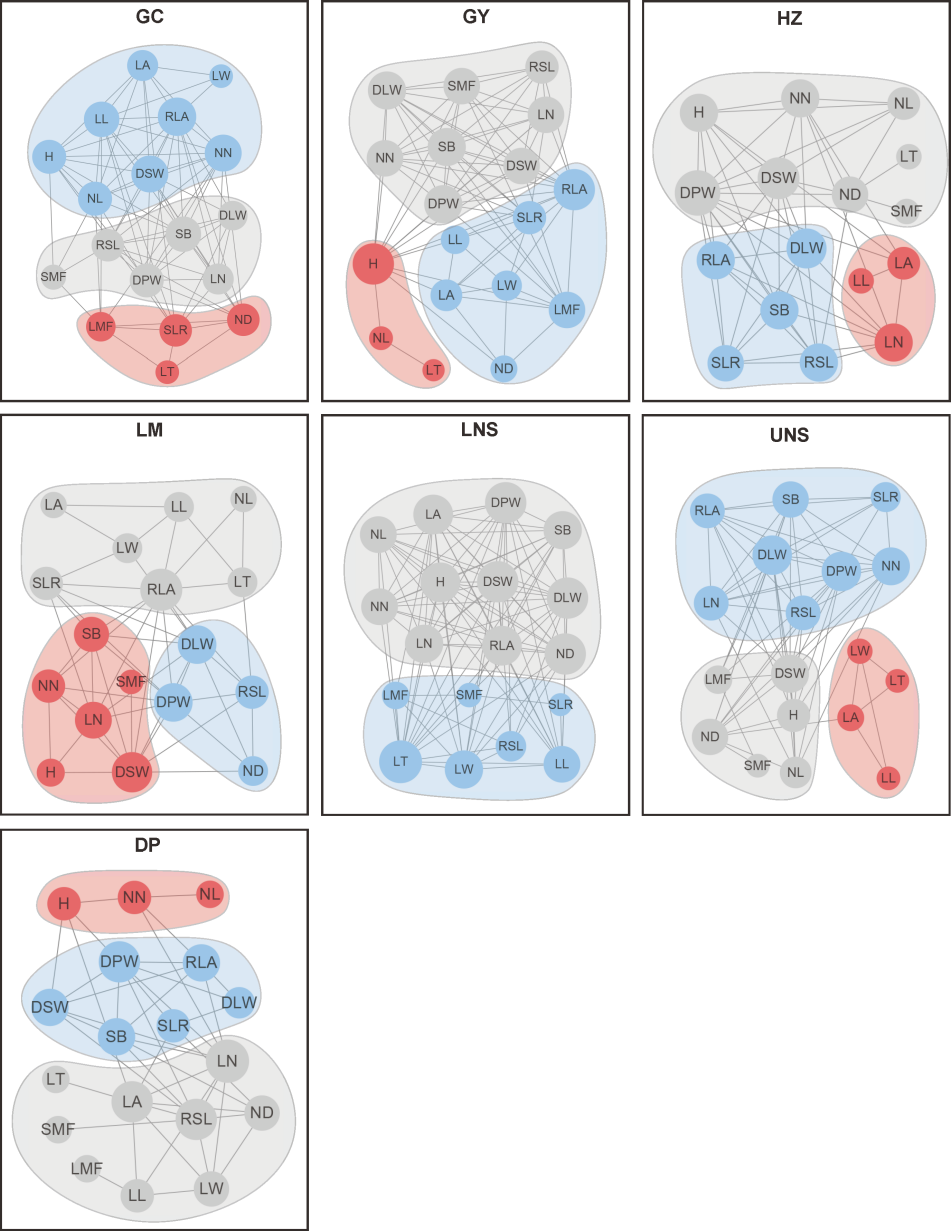
Plant trait network of submerged macrophytes in impounded lakes and Grand Canal. H, Plant height; SB, Stem branch; NN, Internodes number; NL, Internode length; ND, Internode diameter; RSL, Relative stem length; LN, Leaf number; LT, Leaf thickness; LL, Leaf length; LW, Leaf width; LA, Leaf area; RLA, Relative leaf area; DPW, Dry plant weight; DSW, Dry stem weight; DLW, Dry leaf weight; SLR, Ratio of stem weight to leaf weight; SMF, Stem dry mass fraction; LMF, Leaf dry mass fraction.

PTNs showed different structures among impounded lakes and Grand Canal (**Figure 5**). The number of edges within network was highest in lower Nansi Lake and lowest in Dongping Lake (**Figure 6**). A module was a set of plant traits more closely connected to each other but less connected with traits outside the module. In our study, different modules with different colors were observed in the PTNs among impounded lakes and Grand Canal (**Figure 6**). Two modules were detected in PTN in lower Nansi Lake, and three modules were identified for PTNs in others impounded lakes and Grand Canal.

Traits connections in PTNs differed among impounded lakes and Grand Canal. PTNs displayed higher edge density in lower Nansi Lake (0.641) but lower in Dongping Lake (0.275) and Luoma Lake (0.314). Similarly, average clustering coefficient was higher in lower Nansi Lake (0.842) but lower in Dongping Lake (0.453) and Luoma Lake (0.610). Conversely, PTNs exhibited higher average path length and modularity in Dongping Lake (average path length = 2.046 and modularity = 0.227, respectively) and Luoma Lake (average path length = 1.830 and modularity = 0.206, respectively) but lower in lower Nansi Lake (average path length = 1.380 and modularity = 0.067, respectively).

### 3.5 Correlations between PTNs topologies and environmental factors

Pearson correlation showed that average path length significantly related to TP (*r*=-0.84, *p*<0.05), PO_4_^3+^-P (*r*=-0.85, *p*<0.05) and DO (*r*=0.88, *p*<0.05); edge density was closely correlated with TP (*r*=0.87, *p*<0.05) and DO (*r*=-0.86, *p*<0.05); average clustering coefficient significantly correlated with DO (*r*=-0.89, *p*<0.01); modularity significantly related to DO (*r*=0.87, *p*<0.05).

To examine the effects of three significant factors on PTNs average path length, a backward multiple regression analysis was computed. This regression result showed that average path length was negatively correlated to TP, but positively to DO.


(1) $$AL=\;1.22\;-\;24.30TP\;+\;0.15DO\;\left({\text{R}}^{2\;}=0.91;F=19.368;P<0.01\right)$$

To explore the relative role of the two significant factors in explaining the edge density, a backward multiple regression analysis was computed. The result indicated that edge density increases with increasing TP and with reducing DO.


(2) $$ED=\;0.62\;+\;14.59\;TP\;-\;0.07\;DO\;\left({\text{R}}^{2\;}=0.91;F=21.308;P<0.01\right)$$

To reveal the relationships between average clustering coefficient and modularity and DO, a univariate linear regression analysis was performed. The results suggested that DO increases with increasing modularity and with reducing average clustering coefficient.


(3) $$AC=\;1.62\;-0.122\;DO\;\left({\text{R}}^{2\;}=0.80;F=20.12;P<0.01\right)$$

(4) $$Q=\;-0.23+0.05\;DO\;\left({\text{R}}^{2\;}=0.75;F=14.98;P<0.05\right)$$

## 4 Discussion

In this study, we applied a network analysis to identify the hub traits within PTNs, examined the variation of PTNs topologies, and tested the effects of determining factors on the PTNs topologies in impounded lakes and channel river of ERSNWTP. First, we revealed that functional traits significantly differ among impounded lakes and Grand Canal. Next, our results demonstrated that leaf-related traits and organ allocation traits were the hub traits of PTNs in impounded lakes and Grand Canal overall. Furthermore, we identified variability in trait network structures among impounded lakes and Grand Canal. Specifically, tight assemblages of two modules in lower Nansi Lake indicated high-efficiency traits cooperation and resource acquisition. Finally, we clarified the effects of environmental factors on the PTNs. Notably, submerged macrophytes PTNs topologies were determined by TP and DO.

### 4.1 The consistency of the hub traits and sensitive traits

**Figure 7:**
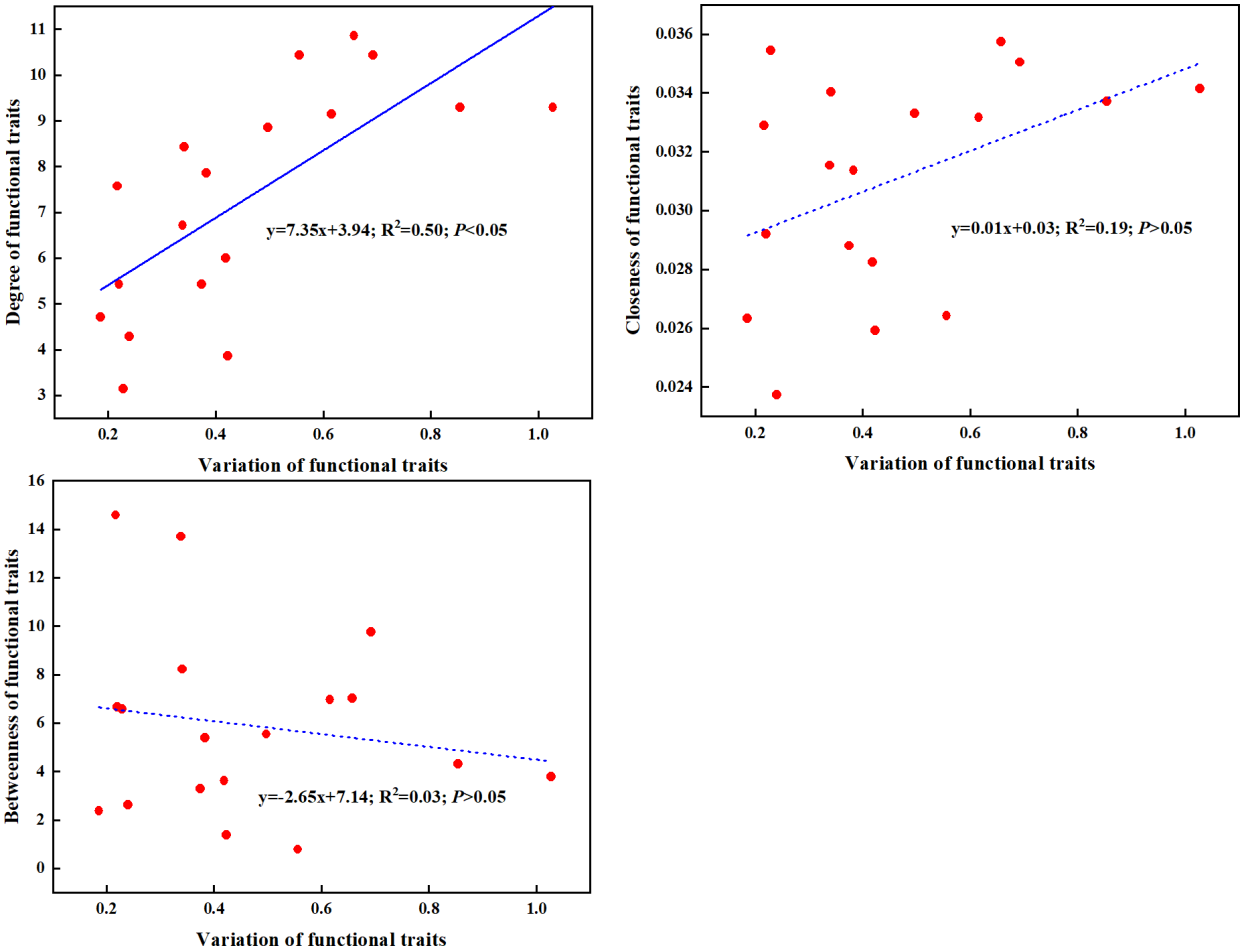
Linear regressions between functional traits variation and degree, closeness and betweenness. Dashed lines indicate that the slope of the linear regression is nonsignificant.

The coefficient of variation of traits showed that stem branch, dry leaf weight, relative leaf area, dry plant weight, leaf number and dry stem weight were more sensitive to varying environment than others traits (Siefert et al., 2015; Yang et al., 2020; Westerband et al., 2021). Noteworthily, these sensitive traits were the hub traits of most PTNs, which indicated that traits with high variability were more likely to be the hub traits of PTNs among impounded lakes and Grand Canal overall. These results can be explained by the finding that functional traits degree displayed significant increase with increasing variation of functional traits (**Figure 7**). Traits with higher degree plays a central role that affects the whole-plant phenotype (Kleyer et al., 2019; He et al., 2020; Li et al., 2021), thus high variability of hub traits makes the trait network more responsive to diverse environments, which may be the reason why the PTNs can be used as an effective approach to clarify functional adaptation of submerged macrophytes to changing environmental conditions.

Although the hub traits of PTNs were different among impounded lakes and Grand Canal, relative leaf area and leaf number were the robust hub traits, which was in agreement with previous study on the forest leaf trait network shifts along latitude gradients, indicating that leaf thickness and relative leaf area were hub traits of leaf trait network (Li et al., 2021). Leaf is the primary organ of submerged macrophytes for light and nutrition resources acquisition (Wright et al., 2004; Shipley et al., 2006; Donovan et al., 2011). Submerged macrophytes tend to increase leaf area, leaf number and relative leaf area to adapt to underwater light limitation (Pierce et al., 2012; Fu et al., 2018; Chmara et al., 2019; Zervas et al., 2019; Liu et al., 2021). Indeed, relative leaf area is a part of the leaf economic spectrum (LES) and closely correlated with other traits related to resource acquisition during plant life history (Price et al., 2014; Reich and Cornelissen, 2014; Díaz et al., 2016).

Moreover, organ mass allocation traits, including dry plant weight, dry stem weight and dry leaf weight, were as central as leaf traits in PTNs. The result was consistent with recent finding that stem- and leaf- traits (stem ratio and leaf ratio) significantly related to biomass allocation were the hub traits in submerged macrophyte PTNs (Yuan et al., 2023). Previous studies revealed that plant height was closely related to stem height and leaf area (Price et al., 2014; Díaz et al., 2016), and the capacity for light and nutrient resource acquisition depends on the allocation of biomass to leaves and stem (Hilbert, 1990; Barko et al., 1991). Notably, shifts in biomass allocation is an important strategy for plants to overcome environmental heterogeneity (McConnaughay and Coleman, 1998; Madsen and Cedergreen, 2002). Submerged macrophytes usually adjust organ mass allocation for optimizing resource capture and minimizing imbalance in any critical environment. (Barko and Smart, 1988; Xie et al., 2010). Thus, organ mass allocation traits as hub traits in our study mainly explained that leaf and stem mass allocation determined light and nutrient resource capture, and shifts in plant biomass allocation happen throughout the whole life of submerged macrophytes (Chambers et al., 1989; Xie et al., 2004).

Furthermore, we noted that hub traits of PTNs are relative to the included traits. A morphological trait is more likely to be a hub if many morphological traits are included in PTNs; likewise, a physiological trait is more likely to be a hub if many physiological traits are included. Traits selection could influence the hub traits of PTNs, to an extent that needs deep study in future work.

### 4.2 Plant trait network topologies and its main influencing factors in impounded lakes

**Figure 8:**
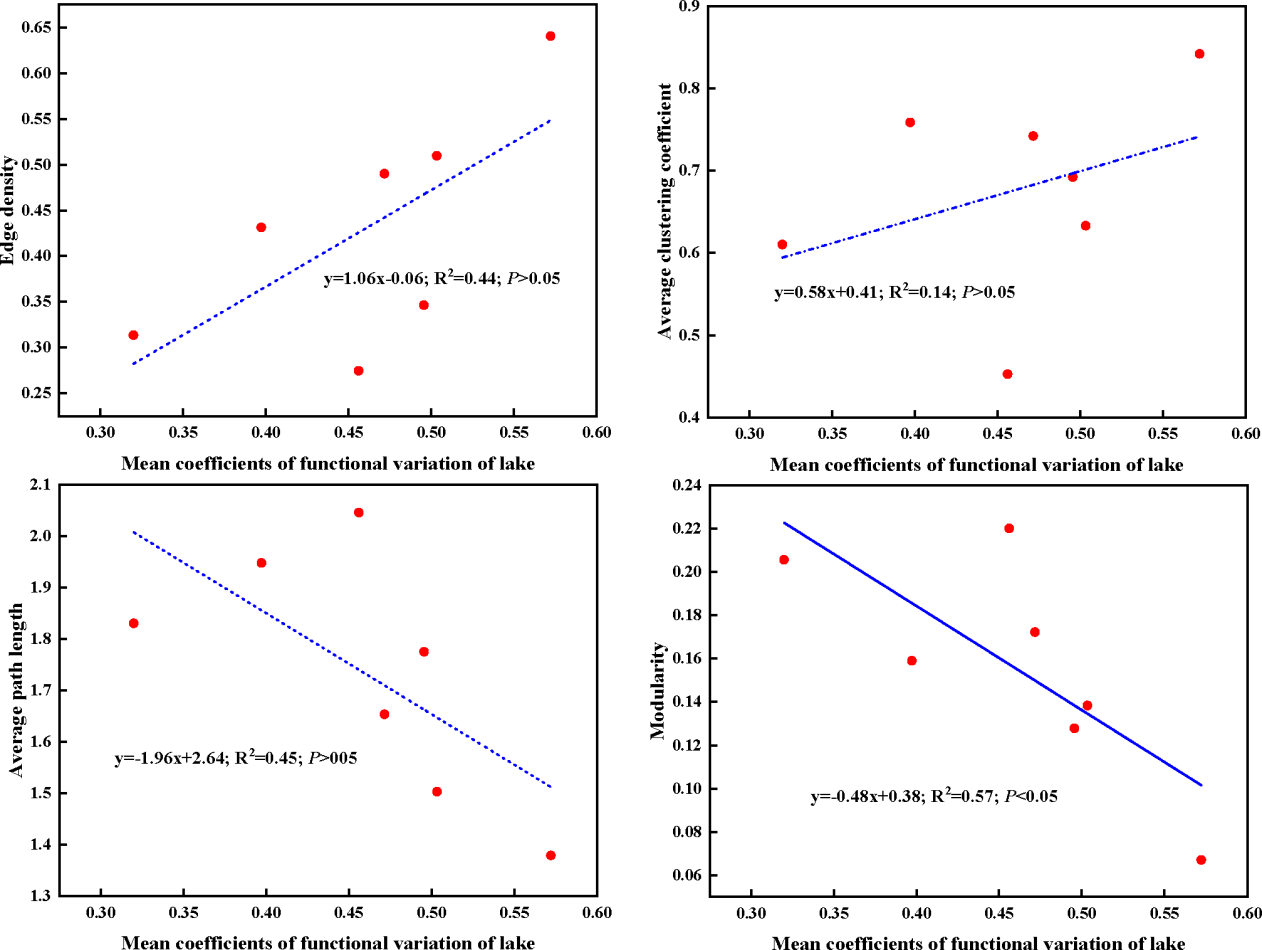
Linear regressions between mean coefficients of functional traits variation of lake and plant trait network parameters. Dashed lines indicate that the slope of the linear regression is nonsignificant.

In present study, higher edge density and average clustering coefficient, whereas lower average path length and modularity of plant trait network were observed in lower Nansi Lake. The opposite trend for these network parameters was detected in Dongping Lake and Luoma Lake. Regarding to the mean CV of traits of impounded lakes and Grand Canal from high to low, the order was lower Nansi Lake, Hongze Lake and Grand Canal, Gaoyou Lake, Dongping Lake, upper Nansi Lake, Luoma Lake (**Table S4**). This finding demonstrated that plant trait network topologies were related to the mean functional trait variations of lakes, higher mean functional variation coefficients represented tight plant trait network, lower mean functional variation coefficients indicated loose plant trait network (**Figure 8**). These results resulted from the significantly negative relationship between mean coefficients of functional variation of lake and modularity of PTNs (**Figure 8**). Obviously, higher mean functional variation coefficients make smaller separation of trait clusters within the network and raises the possibility of functional traits connecting to other traits.

Numerous studies proved that submerged macrophytes would alter multiple functional traits and their relationships in different environmental conditions (Fu et al., 2018; Liu et al., 2021; Wang et al., 2021). In our experiment, water total phosphorus had a significantly positive effect on the edge density and negative effect on the average path length of PTNs, which indicated that trait coordination and integration and thus efficient resource transporting was gradually strong with an increase phosphorus concentration. Contemporary study focusing on the effects of phosphorus on the submerged macrophytes trait network topologies suggested that when water TP was< 0.2 mg/L, edge density increased with increasing TP concentration. In our study, the range of water TP was 0.014 mg/L~0.055 mg/L, the mean water TP was 0.022 mg/L, far less than 0.2 mg/L. Our results were consistent with previous conclusion (Rao et al., 2022). Additionally, dissolved oxygen was another influencing factor determining PTNs topologies. On the contrary, dissolved oxygen had significantly positive effects on average path length and modularity, and negative effects on edge density and average clustering coefficient. Numerous studies demonstrated that aquatic plant tended to elongate leaf length, reduce leaf thickness and decrease overground biomass accumulation in anoxic condition (Kay et al., 2004). Hub traits held the largest connections to others traits and occupied the centre in PTNs (Kleyer et al., 2019; He et al., 2020; Li et al., 2021; Li et al., 2022; Rao et al., 2022). Therefore, an environmental factor such as dissolved oxygen selecting on leaf-related traits and organ mass allocation traits will affect other traits, thus changing the PTNs topology. Thus, both the TP and DO together determine the PTNs topologies in impounded lakes. It should be mentioned that we only studied the unilateral effects of TP and DO, the interactional impact on PTNs topologies need further evaluation.

We found that TP was one of two determined factors influencing the plant trait network of impounded lakes of ERSNWTP. ERSNWTP pumps water from the Yangtze River in Yangzhou, utilizes the Grand Canal and its parallel rivers to transfer water from south to north. For Yangtze river in Jiangsu Province, water total phosphorus concentration ranged from 0.07 to 0.10 mg/L (Chen et al., 2020), which is much higher than the impounded lakes and Grand Canal. Total phosphorus concentration of impounded lakes will increase with the enduring operation of the water diversion, which will result in a strong trait coordination and an efficient resource transporting of submerged macrophytes. Furthermore, future studies should pay more attention on long-term effects of total phosphorus change induced by water transferring on submerged macrophytes PTNs. In addition, more attention should be pay to total phosphorus reduction before submerged macrophyte restoration projects in aquatic ecosystems from PTNs perspective.

## 5 Conclusion

In this study, we applied a network analysis to identify the hub traits and tested the variation of PTNs topologies among impounded lakes and Grand Canal of the ERSNWTP in China. Then, correlations between traits and PTNs topology were examined and determining environmental factors of PTNs were detected using a multivariate statistical analysis. We found that leaf-related traits and organ mass allocation traits were the hub traits of PTNs in impounded lakes and Grand Canal of the ERSNWTP overall. The coefficients variation of functional traits had significantly positive relationships with traits degree, and traits with high variability were more likely to be the hub traits in PTNs. The PTNs exhibited different structures among impounded lakes and Grand Canal, plant trait network topologies were significantly related to the mean functional variation coefficients of lakes. TP and DO were the key factors influencing PTNs topology. TP was significantly related to edge density and average path length, and DO was significantly correlated with edge density and average path length, average clustering coefficient and modularity.

## Data availability statement

The original contributions presented in the study are included in the article/**Supplementary Material**. Further inquiries can be directed to the corresponding author.

## Author contributions

TZ, MZ and QR conceived the study. WJ, HS and TZ performed the field survey and sample collection. Data and statistical analysis was done by TZ and QR. TZ wrote the manuscript and JY, JC, ZG, LW and QR revised it. All authors contributed to the article and approved the submitted version.
