# Supplementary material for: Characteristics of plant trait network and its influencing factors in impounded lakes and channel rivers of South-to-North Water Transfer Project, China
Source: Front Plant Sci. 2023 Mar 10;14:1127209. doi: 10.3389/fpls.2023.1127209 (PMC10036390; doi:10.3389/fpls.2023.1127209)
Supplement: Supplementary file 1 [file DataSheet_1.docx]

**Table S1 The morphometrical and limnological characteristics of five impounded lakes of East Route of South to North Water Transfer Project**

| **Parameters** | **Gaoyou Lake** | **Hongze Lake** | **Luoma Lake** | **Nansi Lake** | **Dongping Lake** |
| --- | --- | --- | --- | --- | --- |
| Latitude | 32°42′~33°04′N | 33°06′~33°40′N | 34°00′~34°14′N | 34°27′~35°20′N | 35°43′~36°07′N |
| Longitude | 119°06′~119°25′E | 118°10′~118°52′E | 118°04′~118°18′E | 116°34′~117°21′E | 116°02′~116°20′E |
| Mean water depth (m) | 1.4 | 1.8 | 3.3 | 1.5 | 2.0 |
| Average annual air temperature (℃) | 14.7 | 14.5 | 14.1 | 13.0 | 13.4 |
| Surface area (km^2^) | 675 | 1597 | 290 | 1266 | 209 |
| Storage capability (m^3^) | 9.7×10^8^ | 27.9×10^8^ | 9.2×10^8^ | 16.1×10^8^ | 7.8×10^8^ |

**Table S2 Variation of environmental factors among impounded lakes/Grand Canal (mean±SE)**

| **Environmental factors** | **GC** | **GY** | **HZ** | **LM** | **UNS** | **LNS** | **DP** |
| --- | --- | --- | --- | --- | --- | --- | --- |
| Cond（µS cm^-1^） | 894.41±85.89c | 384.81±10.94f | 479.07±12.62ef | 583.08±12.04de | 1077.20±25.71b | 627.74±30.29d | 1273.69±14.25a |
| pH | 8.08±0.08c | 8.26±0.09bc | 8.07±0.09c | 8.20±0.15bc | 8.36±0.10bc | 8.58±0.26b | 8.96±0.13a |
| DO（mg L^-1^） | 7.55±0.63bc | 7.35±0.15bc | 7.66±0.14b | **7.82±0.34b** | 7.89±0.34b | 6.22±0.69c | **9.34±0.41a** |
| TDS（mg L^-1^） | 626.99±65.77c | 305.99±7.67e | 385.78±10.43de | 454.15±9.66d | 751.49.15±19.18b | 457.51±18.12d | 1011.32±11.53a |
| Tur（FNU） | 58.84±20.84c | 79.52±22.62bc | 173.45±52.34ab | 208.85±61.89a | 206.68±29.95a | 224.07±60.38a | 63.70±12.46c |
| SD（cm） | 59.64±7.21b | 31.73±3.45c | 29.38±1.75c | 148.89±12.96a | 145.00±15.95a | 79.38±5.13b | 66.50±7.19b |
| *K*（m^-1^） | 2.23±0.17c | 3.47±0.29a | 2.91±0.19ab | 0.76±0.09d | 1.07±0.13d | 0.81±0.02d | 2.62±0.22bc |
| COD_Mn_（mg L^-1^） | 2.18±0.27a | 2.22±0.34a | 1.71±0.09a | 1.57±0.06a | 2.20±0.04a | 2.32±0.10a | 2.03±0.07a |
| Chl *a*（μg L^-1^） | 9.15±1.38bc | 21.84±3.72a | 15.26±2.35ab | 5.34±0.93c | 7.33±1.09c | 6.84±1.73c | 5.51±0.65c |
| TN（mg L^-1^） | 1.23±0.16c | 1.89±0.16a | 1.67±0.10ab | 1.41±0.13bc | 0.62±0.04d | 1.43±0.09bc | 0.54±0.01d |
| NO_3_^-^-N（mg L^-1^） | 0.98±0.18b | 1.66±0.18a | 1.49±0.10a | 0.95±0.13b | 0.11±0.02c | 0.96±0.11b | 0.07±0.01c |
| NH_4_^+^- N（mg L^-1^） | 0.05±0.01b | 0.05±0.01b | 0.03±0.00b | 0.16±0.02a | 0.18±0.02a | 0.18±0.03a | 0.14±0.01a |
| TP（mg L^-1^） | 0.03±0.00a | 0.03±0.00bc | 0.02±0.00c | 0.02±0.00c | 0.02±0.00bc | 0.03±0.00ab | **0.02±0.00c** |
| PO_4_^3+^-P（mg L^-1^） | 0.02±0.00a | 0.02±0.00ab | 0.02±0.00bc | 0.01±0.00c | 0.01±0.00bc | 0.02±0.00ab | 0.01±0.00c |
| S_w_（%） | 37.27±3.38c | 52.00±2.35b | 45.62±3.53bc | 56.23±4.94ab | 67.92±3.62a | 64.86±6.19a | 47.71±4.64bc |
| S_o_（%） | 5.49±1.04ab | 8.03±2.63ab | 4.66±0.32b | 5.86±0.66ab | 10.64±1.76a | 8.52±1.35ab | 7.62±0.76ab |
| S_C_（mg g^-1^） | 34.21±2.05c | 46.03±3.20bc | 45.03±4.48bc | 57.32±6.10b | 78.11±9.32a | 82.80±5.56a | 57.11±5.20b |
| S_N_（mg g^-1^） | 5.04±0.58d | 6.24±0.43cd | 6.18±0.49cd | 7.15±0.59bcd | 9.09±1.38ab | 9.62±0.80a | 8.16±0.84abc |
| S_P_（mg g^-1^） | 1.16±0.07a | 1.27±0.13a | 1.21±0.07a | 1.04±0.06a | 1.14±0.07a | 1.16±0.04a | 1.31±0.06a |

T: Temperature; Cond: Conductivity; DO: Dissolved oxygen; TDS: Total dissolved solids; Tur: Turbidity; SD: Transparency; *K*: Extinction coefficient; COD_Mn_: Permanganate index; Chl *a*: chlorophyll *a*; TN: Total nitrogen; NO_3_^-^-N: Nitrate nitrogen; NH_4_^+^-N: Ammonia nitrogen; TP: Total phosphorus; PO_4_^3+^-P: Orthophosphate; S_W_: Sediment water content; S_O_: Sediment organic matter content; S_C_: Total sediment carbon content; S_N_: Total sediment nitrogen content; S_P_: Total sediment phosphorous content. Different letters indicated the significance according to Duncan’s test at the 0.05 level

**Table S3 Variation of functional traits among impounded lakes/Grand Canal (mean±SE)**

| **Functional traits** | **GC** | **GY** | **HZ** | **LM** | **UNS** | **LNS** | **DP** |
| --- | --- | --- | --- | --- | --- | --- | --- |
| H (cm) | 93.35±5.91e | 179.55±9.39cd | 230.23±16.44b | 263.99±16.60a | 208.18±6.36bc | 122.73±13.49e | 171.91±7.72d |
| SB | 7.57±0.90cd | 10.38±0.99bc | 12.79±1.51ab | 14.74±1.11a | 6.23±0.98d | 1.54±0.74e | 2.37±0.69e |
| NN | 45.31±4.62b | 30.93±1.40b | 69.17±10.07a | 35.78±1.72b | 65.20±7.53a | 31.46±3.94b | 32.20±1.87b |
| NL (cm) | 5.09±0.39d | 6.76±0.42bc | 10.08±0.71a | 8.90±0.64a | 7.36±0.32b | 5.52±0.56cd | 7.01±0.33b |
| ND (mm) | 1.59±0.06ab | 1.18±0.06d | 1.63±0.09a | 1.44±0.05bc | 1.65±0.04a | 1.30±0.06cd | 1.42±0.04bc |
| RSL (cm g^-1^) | 520.79±31.18b | 525.71±37.82b | 507.52±49.40bc | 398.15±31.48c | 715.16±43.23a | 726.03±42.39a | 756.65±41.53a |
| LN | 83.07±8.20b | 117.13±10.38a | 117.71±13.32a | 135.70±8.26a | 75.10±10.76b | 28.71±4.68c | 42.97±4.97c |
| LT (mm) | 0.56±0.05b | 0.40±0.03cd | 0.56±0.06b | 0.50±0.04bc | 0.45±0.02bcd | 0.34±0.02d | 0.69±0.06a |
| LL (cm) | 4.53±0.18cd | 5.19±0.21ab | 4.69±0.25bc | 5.49±0.26a | 5.05±0.15abc | 4.07±0.15d | 4.93±0.14abc |
| LW (cm) | 0.82±0.02c | 0.86±0.02bc | 0.87±0.04bc | 1.05±0.03a | 0.92±0.03b | 0.73±0.03d | 0.80±0.03cd |
| LA (cm^2^) | 3.01±0.16c | 3.71±0.24b | 3.00±0.23c | 4.61±0.29a | 3.90±0.20b | 2.51±0.17c | 3.03±0.15c |
| RLA (cm^2^ g^-1^) | 13.17±1.29c | 12.01±1.49c | 10.31±2.29c | 7.60±0.76c | 19.48±2.17b | 31.12±3.49a | 30.22±3.37a |
| DPW (g) | 0.59±0.06cd | 0.97±0.10b | 1.44±0.23a | 1.46±0.10a | 0.74±0.08bc | 0.41±0.08d | 0.55±0.05cd |
| DSW (g) | 0.21±0.02e | 0.43±0.04c | 0.55±0.07b | 0.71±0.04a | 0.34±0.03cd | 0.18±0.03e | 0.24±0.02de |
| DLW (g) | 0.35±0.05d | 0.52±0.06c | 0.68±0.12ab | 0.72±0.07a | 0.30±0.04de | 0.13±0.03e | 0.16±0.04e |
| SLR | 0.74±0.07d | 1.02±0.08c | 0.96±0.09cd | 1.09±0.07c | 1.36±0.10b | 1.44±0.08b | 1.81±0.10a |
| SMF | 0.09±0.00c | 0.11±0.00ab | 0.09±0.00c | 0.10±0.00bc | 0.07±0.00d | 0.09±0.00c | 0.11±0.00a |
| LMF | 0.18±0.01a | 0.17±0.01a | 0.19±0.01a | 0.18±0.01a | 0.13±0.01b | 0.14±0.01b | 0.19±0.01a |

GC: Grand Canal; GY: Gaoyou Lake; HZ: Hongze Lake; LM: Luoma Lake; UNS: upper Nansi Lake; LNS: lower Nansi Lake; DP: Dongping Lake; H: plant height; SB: stem branch; NN: internodes number; NL: internode length; ND: internode diameter; RSL: relative stem length; LN: leaf number; LT: leaf thickness; LL: leaf length; LW: leaf width; LA: leaf area; RLA: relative leaf area; DPW: dry plant weight; DSW: dry stem weight; DLW: dry leaf weight; SLR: ratio of stem weight to leaf weight; SMF: stem dry mass fraction; LMF: leaf dry mass fraction. Different letters indicated the significance according to Duncan’s test at the 0.05 level

**Table S4 Coefficients of variation of functional traits among channel river and impounded lakes**

| **Functional traits** | **GC** | **GY** | **HZ** | **LM** | **UNS** | **LNS** | **DP** | **Mean CV of trait** |
| --- | --- | --- | --- | --- | --- | --- | --- | --- |
| H (cm) | 0.40 | 0.35 | 0.35 | 0.33 | 0.17 | 0.54 | 0.25 | 0.34±0.04 |
| SB | 0.77 | 0.64 | 0.58 | 0.39 | 0.86 | 2.35 | 1.60 | 1.03±0.26 |
| NN | 0.66 | 0.30 | 0.71 | 0.25 | 0.63 | 0.61 | 0.32 | 0.50±0.07 |
| NL (cm) | 0.49 | 0.41 | 0.35 | 0.37 | 0.24 | 0.50 | 0.26 | 0.37±0.04 |
| ND (mm) | 0.25 | 0.35 | 0.28 | 0.16 | 0.13 | 0.21 | 0.14 | 0.22±0.03 |
| RSL (cm g^-1^) | 0.39 | 0.48 | 0.48 | 0.41 | 0.33 | 0.29 | 0.30 | 0.38±0.03 |
| LN | 0.64 | 0.59 | 0.55 | 0.32 | 0.78 | 0.80 | 0.63 | 0.62±0.06 |
| LT (mm) | 0.55 | 0.53 | 0.51 | 0.42 | 0.26 | 0.23 | 0.46 | 0.42±0.05 |
| LL (cm) | 0.26 | 0.28 | 0.26 | 0.24 | 0.16 | 0.18 | 0.16 | 0.22±0.02 |
| LW (cm) | 0.17 | 0.19 | 0.20 | 0.15 | 0.18 | 0.19 | 0.22 | 0.19±0.01 |
| LA (cm^2^) | 0.35 | 0.43 | 0.38 | 0.32 | 0.28 | 0.34 | 0.27 | 0.34±0.02 |
| RLA (cm^2^ g^-1^) | 0.64 | 0.83 | 1.09 | 0.52 | 0.61 | 0.55 | 0.61 | 0.69±0.08 |
| DPW (g) | 0.71 | 0.68 | 0.79 | 0.35 | 0.60 | 0.96 | 0.51 | 0.66±0.07 |
| DSW (g) | 0.64 | 0.64 | 0.59 | 0.32 | 0.51 | 0.85 | 0.34 | 0.56±0.07 |
| DLW (g) | 0.85 | 0.79 | 0.87 | 0.48 | 0.73 | 1.00 | 1.26 | 0.85±0.09 |
| SLR | 0.60 | 0.55 | 0.46 | 0.31 | 0.41 | 0.28 | 0.32 | 0.42±0.05 |
| SMF | 0.37 | 0.20 | 0.23 | 0.24 | 0.12 | 0.22 | 0.22 | 0.23±0.03 |
| LMF | 0.32 | 0.25 | 0.24 | 0.18 | 0.15 | 0.20 | 0.34 | 0.24±0.03 |
| Mean CV of lake | 0.50±0.05 | 0.47±0.05 | 0.50±0.06 | 0.32±0.03 | 0.40±0.06 | **0.57±0.12** | 0.46±0.09 |  |

GC: Grand Canal; GY: Gaoyou Lake; HZ: Hongze Lake; LM: Luoma Lake; UNS: upper Nansi Lake; LNS: lower Nansi Lake; DP: Dongping Lake; H: plant height; SB: stem branch; NN: internodes number; NL: internode length; ND: internode diameter; RSL: relative stem length; LN: leaf number; LT: leaf thickness; LL: leaf length; LW: leaf width; LA: leaf area; RLA: relative leaf area; DPW: dry plant weight; DSW: dry stem weight; DLW: dry leaf weight; SLR: ratio of stem weight to leaf weight; SMF: stem dry mass fraction; LMF: leaf dry mass fraction
